# Integrating orientation mechanisms, adrenocortical activity, and endurance flight in vagrancy behaviour

**DOI:** 10.1038/s41598-022-26136-8

**Published:** 2022-12-21

**Authors:** Katherine R. S. Snell, Rebecca C. Young, Jesse S. Krause, J. Martin Collinson, John C. Wingfield, Kasper Thorup

**Affiliations:** 1 Center for Macroecology, Evolution and Climate, Natural History Museum of Denmark, University of Copenhagen, Universitetsparken 15, 2100 Copenhagen, Denmark; 2 Biological Sciences, North Dakota State University, P.O. Box 6050, Fargo, ND 58108 USA; 3 Department of Neurobiology, Physiology and Behaviour, University of California, One Shields Avenue, Davis, CA 95616 USA; 4 School of Medicine, Medical Sciences and Nutrition, Institute of Medical Sciences, University of Aberdeen, Aberdeen, AB25 2ZD UK; 5 Present Address: Max-Planck Institute of Animal Behavior, Am Obstberg 1, 78315 Radolfzell, Germany

**Keywords:** Hormones, Ecology, Animal migration, Ecophysiology

## Abstract

Avian migratory processes are typically precisely oriented, yet vagrants are frequently recorded outside their normal range. Wind displaced vagrants often show corrective behaviour, and as an appropriate response is likely adaptive. We investigated the physiological response to vagrancy in passerines. Activation of the emergency life-history stage (ELHS), assessed by high baseline plasma corticosterone, is a potential mechanism to elicit compensatory behaviour in response to challenges resulting from navigational error, coupled with response to fuel load and flight. We compared circulating plasma corticosterone concentrations and body condition between three migratory groups in autumn: (1) wind displaced southwest (SW) vagrants and (2) long range southeast (SE) vagrants on the remote Faroe Islands, and (3) birds within the expected SW migratory route (controls) on the Falsterbo peninsula, Sweden. Vagrants were further grouped by those sampled immediately upon termination of over-water migratory flight and those already on the island. In all groups there was no indication of the activation of the ELHS in response to vagrancy. We found limited support for an increased rate of corticosterone elevation within our 3 min sample interval in a single species, but this was driven by an individual ELHS outlier. Fat scores were negatively correlated with circulating corticosterone; this relationship may suggest that ELHS activation depends upon an individual’s energetic states. Interestingly, in individuals caught at the completion of an obligate long-distance flight, we found some evidence of corticosterone suppression. Although limited, data did support the induction of negative feedback mechanisms that suppress corticosterone during endurance exercise, even when fuel loads are low.

## Introduction

**Figure 1:**
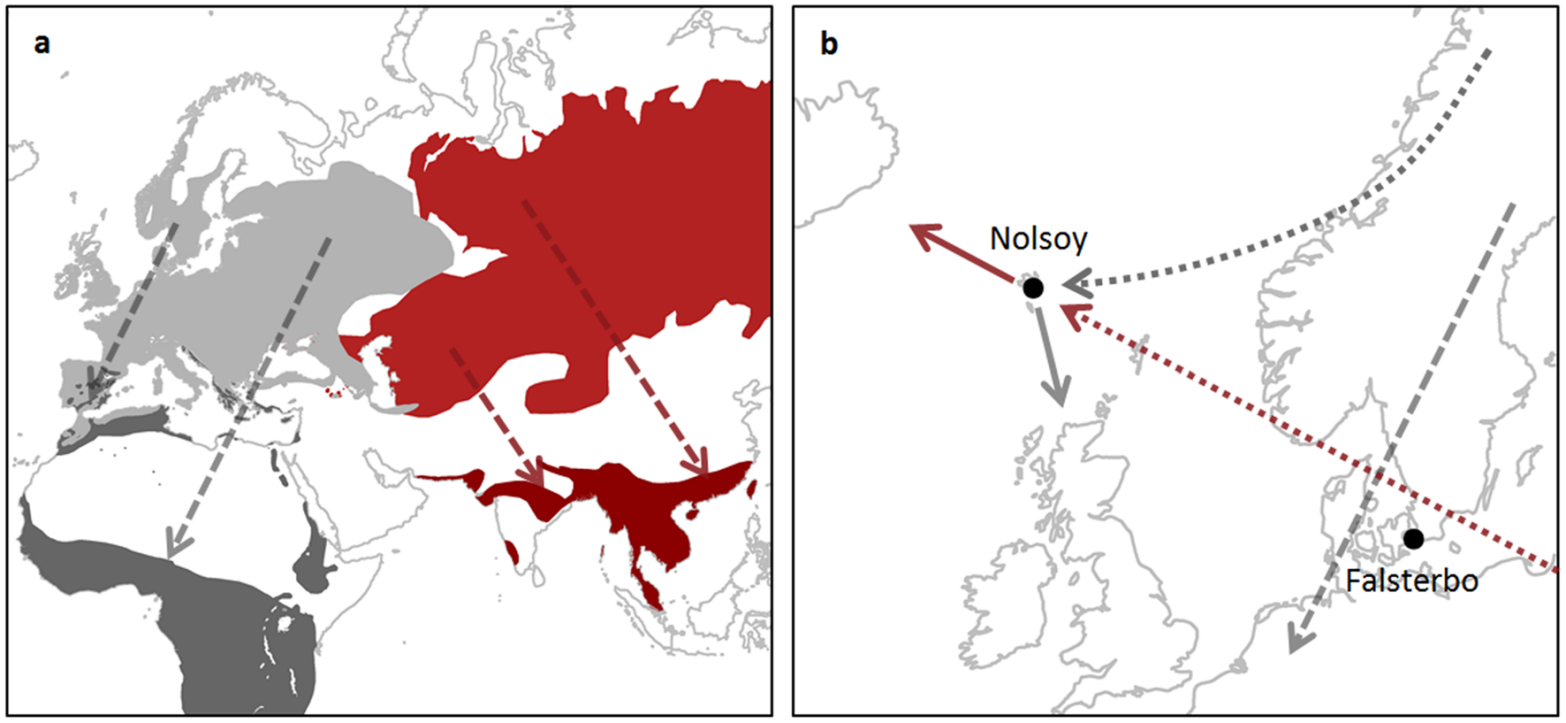
(**a**) The breeding (dark shading) and wintering (light shading) sites of SW vagrant (grey) and SE vagrant (red) species. Blackcap and garden warbler ranges (SW vagrants) and yellow-browed warbler and paddyfield warbler (SE vagrants) are depicted here for illustration. Range data from BirdLife International and Handbook of the Birds of the World^12^ and map drawn in R (version 3.5.3,^13^). Dashed lines represent the autumn migration standard orientation of the study species. (**b**) Sampling sites. Faroe Islands for southwest (SW) vagrant and southeast (SE) vagrant birds; the location of Nólsoy (one of the most eastern islands of the Faroe archipelago, where birds were captured) and Falsterbo Bird Observatory (situated at the southern tip of Sweden) for SW controls. The dashed line represents the autumn migration standard orientation of SW species of which controls were sampled at Falsterbo; dotted lines represent the expected routes the migratory groups travel to be encountered on the Faroe Islands SW vagrant (grey) and SE vagrant (red) species; and short solid lines indicate the expected departure orientation of the two vagrant groups encountered on the Faroe Islands, following Thorup et al.^14^.

Seasonal avian migration ultimately occurs in response to resource availability^1,2^. Approximately 40% of Nearctic and 23% of all Western Palearctic species, equivalent to billions of individuals, embark on considerable annual migrations^3^. Even small passerines have the capacity to navigate over continental scales and undertake non-stop flights that far exceed the duration of one night’s migration^4,5^. Migratory routes between breeding sites and wintering grounds are generally highly conserved within species and populations^1^. In addition, seasonally appropriate migratory orientation is generally population-specific^1,6^. However, instances of aberrant behaviour, the occurrence of individuals far outside their expected range, are recorded annually, and (at least in passerines) these are predominantly young birds undertaking their first autumn migration^7,8^. Birds found outside their normal migratory route, are classified as vagrants; and although scarce, can provide a valuable model for studying migratory processes, including orientation mechanisms and the limitations of endurance flight^8^. In the Western Palearctic, during autumn migration a southwest (SW) standard orientation predominates for birds breeding in Scandinavia, which normally winter in Western Continental Europe or North Africa(^9^; Fig. 1a). Due to wind, a north Scandinavian vagrant may become naturally displaced from its normal SW route and find itself over the Atlantic, where it might land on Iceland, the Faroe Islands, or the British Isles (Fig. 1b). In comparison, misdirected long range vagrants, distinguishable by originating from Asian breeding sites and normally migrating southeast (SE) to winter at sites on the Asian subcontinent (Fig. 1a), may also be encountered in the Western Palearctic(^9–11^; Fig. 1b). Furthermore, occurrences of these rare SE vagrants are apparently concentrated on small island groups such as Shetland Islands, (United Kingdom), the Azores, and the Faroe Islands.

The orientation mechanisms in nocturnal avian migrants are far from being fully explained. However, following extensive experimental and descriptive studies, it is generally accepted that the migratory orientation of young birds is controlled by an innate genetically inherited programme^15–17^. Further to this, Western Palearctic birds (with a SW standard orientation) geographically displaced experimentally or by wind to the Faroe Islands, which is at the periphery of their migratory range, appear to compensate by overwhelmingly demonstrating corrective orientation(^14,18^; Fig. 1b). By stark contrast, SE vagrant species originating from far eastern regions, also tested on the Faroe Islands, failed to correct orientation, departing from the Faroe Islands in a continuation of their misoriented flight(^14^; Fig. 1b). Thus, a compelling argument could be made that SW vagrants are aware of their misdirected flight, while SE vagrants are not. We propose that knowledge of misdirected flight would result in psychological stress resulting a cascade of acute physiological changes which may aid reorientation. We utilised the same study system on the Faroe Islands to quantify the physiological stress response in these rare cohorts of vagrants.

One endocrine system that is consistently activated in response to psychological or physiological challenges is the hypothalamic–pituitary–adrenal (HPA) axis, which controls the synthesis of glucocorticoids to modify physiology and behaviour to help an animal respond to a challenge^19^. During periods of predictable conditions, modulation of baseline glucocorticoid levels are thought to be primarily involved in regulating metabolism, immune function, behaviour, etc.^20^. Glucocorticoid levels fluctuate seasonally based on energetic demands. Specifically, long-distance endurance flight is associated with an extremely high metabolic rate in comparison with other forms of animal locomotion, often occurs while fasting, and coincides with elevations in glucocorticoids^21,22^. Increased circulating corticosterone during exercise liberates glucose and fat from storage sites to fuel higher energy demands^23,24^. Within this range (reported mean values for migrating European passerines ca. 3–22.2 ng ml^−1^; *cit.*), variation has been correlated to migratory readiness^25–27^, fuel load^28^ and fuelling rate^25,29^. During unpredictable events, such as when an individual is challenged by a diverse range of acute stressors (e.g., storm, predator, social challenge, exertion, food deprivation etc.), corticosterone is rapidly synthesised, resulting in a many-fold increase in circulating concentrations, which in turn elicits beneficial adaptive behaviours^20,30,31^. High concentrations of corticosterone (reported mean values for European passerines ca. 15–131.9 ng ml^−1^; *cit.*) is the signature of the emergency life history stage (ELHS), which diverts resources from normal seasonally appropriate daily activity into increased foraging, liberating metabolic fuel, irruptive escape flights, and preparative immune response^22,32,33^. In addition to an elevation of baseline corticosterone in birds undertaking endurance flight, observations of ELHS levels have been reported^23,24^. Thus, the HPA axis is a prime candidate for understanding the potential endocrine basis for coping with natural displacement during migration. We hypothesised that an ELHS underlies the mechanism aiding with navigational correction and/or enduring obligate sustained flight through subsequent physiological and behavioural changes.

We use vagrant passerines captured on the remote archipelago of the Faroe Islands, 500 km west of the Scandinavian Peninsula, as a natural experiment to investigate the adrenocortical response in aberrant migration. Except for a few species breeding on Iceland (excluded in this study), all migrant passerines on the Faroes are outside their normal migration route and can as such be classified as vagrants^34^. These can be divided into two distinct groups by species (Fig. 1), as previously defined in the literature (e.g.^14^): SW vagrants, individuals that have been naturally displaced, having under-corrected for wind drift over a relatively small regional scale^14^; and SE vagrants, individuals migrating with a consistent, directed, but misoriented heading that has been maintained over thousands of kilometres (e.g.,^10,11,35^).

**Figure 2:**
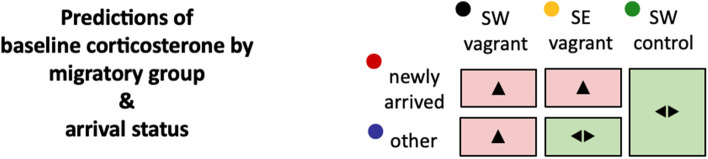
Schematic of predictions of corticosterone concentrations by migratory group and arrival status. Plasma corticosterone was measured in three groups: naturally displaced migrants on the Faroe Islands (SW vagrants); vagrants originating from Asia that have followed a continental scale erroneous course without compensatory behaviour and thus were encountered on the Faroe Islands (SE vagrants); and control southwesterly Scandinavian migrants captured on the migratory flyway in Sweden (SW controls). We predict that high concentrations of circulating corticosterone (of a magnitude consistent with expression of an ELHS) would be present in the naturally displaced group encountered on the Faroes archipelago, but not in control conspecifics on the normal route, nor in the SE vagrant group that have arrived on the Faroes with no apparent perception of being outside their normal range (Fig. 1). Furthermore, in birds captured on the Faroe Islands we investigated the difference in corticosterone concentrations between birds that had just ended a migratory flight with those that had been present for some time. All study individuals on the Faroe Islands had undertaken an estimated 10–15 h of continuous flight (Fig. 1b), and we predict that forced long migratory flight in any bird, regardless of its origin, would vastly increase adrenocortical activity at the time of arrival.

Heightened adrenocortical reactivity may be beneficial for animals that experience a wide variation in environmental conditions (e.g., migrants, high latitude species or populations at the periphery of their ranges), enabling an enhanced responsiveness to challenges^36^. We hypothesised that such a physiologically driven change in behaviour is adaptive in two specific critical circumstances. We tested two predictions of high corticosterone concentrations in critical scenarios: (i) following natural displacement outside the normal migration route, linked to expected subsequent reorientation; or (ii) obligatory long, overwater, migratory flights. Either of these situations may elicit highly elevated baseline circulating corticosterone and initiate the ELHS to promote behavioural changes required to promote corrective reorientation or facilitate refuelling behaviour, respectively (Fig. 2).

## Methods

### Study sites

Both SW vagrants and SE vagrants were caught on Nólsoy (62.00°N, 6.40°W; Fig. 1b), one of the easternmost islands of the Faroes archipelago, with mist-nets (September 2016; capture rate 0.102 h^−1^). This island attracts a large proportion of newly arrived migrants to the Faroe Islands, in part due to the topography of Nólsoy and the small village situated midway between high, barren ground to the north and south, effectively funnelling birds towards suitable cover in the village. This village has also been the site of a long-term monitoring Heligoland trap since 1994^34^.

SW control birds were caught during standardised mist-netting at Falsterbo Bird Observatory (55.38°N, 12.82°E; Fig. 1b) on the southern tip of Sweden during autumn (2015–2017; capture rate 0.031 h^−1^). Here, birds are migrating along the Scandinavia – Northern Europe autumn flyway, having been funnelled down the Scandinavian Peninsula. South of Falsterbo, birds were anticipated to cross the Baltic Sea towards their wintering grounds in Europe or Africa.

### Species, migratory groups and arrival status

**Figure 3:**
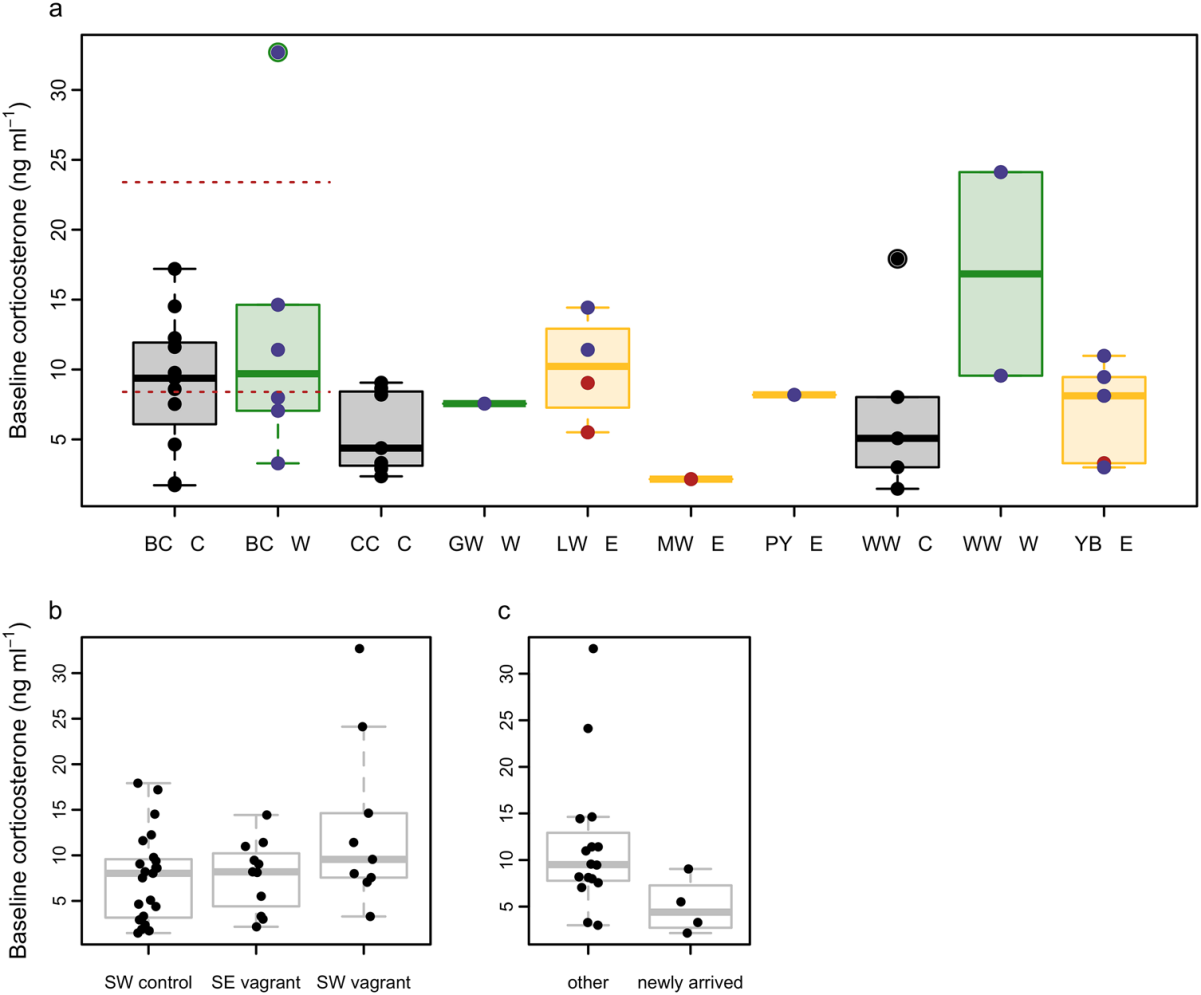
(**a**) All corticosterone concentration measures (ng ml^−1^) depicted by species (BC: blackcap, CC: chiffchaff, GW: garden warbler, LW: lesser whitethroat, MW: marsh warbler, PY: paddyfield warbler, WW: willow warbler and YB: yellow-browed warbler) and migratory group (green C: SW controls from Falsterbo, grey W: SW vagrants and yellow E: SE vagrants). Newly arrived individuals are depicted by (red color) and others by (violet color). The box plots represent the median value and upper and lower quartiles. Whiskers represent the 95% confidence interval and outliers are shown by dots. Red horizontal dotted lines represent the mean baseline (lower) and stress-induced (upper) autumn corticosterone measures of blackcaps sampled on migration reported by Tsvey et al.^42^. Sample sizes: BC C (n = 11), BC W (n = 6), CC C (n = 7), GW W (n = 1), LW E (n = 4), MW E (n = 1), PY E (n = 1), WW C (n = 5), WW W (n = 2), YB E (n = 5). (**b**) Corticosterone concentration measures (ng ml^−1^) by migratory group. Sample sizes: SW control (n = 23), SE vagrants (n = 11), SW vagrants (n = 9) (**c**) Corticosterone concentration measures (ng ml^−1^) from the Faroe by arrival status. Sample sizes: newly arrived (n = 4) and all other (n = 16).

Species included in this study were both common southwesterly flying Scandinavian migrants that were routinely caught at Falsterbo Bird Observatory in autumn (SW controls) and likely naturally wind displaced to the Faroe Islands *en route* from breeding grounds in Northwest Europe (SW vagrants;^34^), and eastern species far outside their normal migration range with breeding grounds many thousands of kilometres distant and considered vagrants when encountered on the Faroe Islands (SE vagrants). Vagrancy is a rare phenomenon and we expected low sample sizes (see Fig. 3), as such we did not restrict sampling to particular species from the outset. We obtained samples from species considered SW vagrants (n = 9): blackcap (*Sylvia atricapilla)*, willow warbler (*Phylloscopus trochilus)* and garden warbler (*Sylvia borin*) and SE vagrants (n = 11): marsh warbler (*Acrocephalus palustris),* paddyfield warbler (*Acrocephalus Agricola),* yellow-browed warbler (*Phylloscopus inoratus)* and the Siberian subspecies of lesser whitethroat (*Sylvia curruca blythi*). For the SW vagrants, the Scandinavian breeding populations winter in southwestern Europe and sub-Saharan Africa; in contrast, SE vagrants have a southeasterly autumn migration direction and typically overwinter on the Asian subcontinent^9,37^. The SW control species (n = 23) sampled in Falsterbo were blackcap, willow warbler and chiffchaff (*Phylloscopus collybita*). All species included are relatively small songbirds with average mass and wing length ranges of 5–22 g and 55–80 mm, respectively. Ageing criteria followed Svensson^38^, and the Siberian lesser whitethroats were confirmed to subspecies by sequencing *cytb* from erythrocytes using the primers L14993 and H16065, as described by Helbig and Seibold^39^, and comparing to birds of known provenance^40,41^. The few birds, diverse species range, and little cover on the island allowed reliable determination of the arrival dates of birds to the Faroe Islands. For arrival status, we classed birds as ‘newly arrived’ migrants only when apparently caught out of directed flight from the Atlantic Ocean to the east, at a time representative of seasonally appropriate migration from the nearest seasonally appropriate coastlines *ca.* 500 km distant (1000–1300 local time). Birds that were known or presumed to be present on the island, were captured arriving from the west or at an inappropriate time were classed as ‘other’. These controls also included birds captured and ringed as part of our program, from which blood was sampled only after a subsequent capture. Our understanding of arrival status is based on extensive field experience and long-term monitoring of sites across the Faroe Islands.

### Physiology and biometrics

Baseline values of circulating corticosterone are difficult to obtain, as hormone concentration increases rapidly in response to capture and handling^31^ and is likely to be species- and season-specific. Generally, statistically measurable changes occur after 3 min, but have been observed as early as 1.5 min in some species^31^. To obtain baseline samples (circulating corticosterone in the absence of handling stress), nets were monitored continuously, and a blood sample (ca. 50–100 µl and < 10% blood volume by body mass^43^) was collected from an alar vein puncture (27 G needle) into a heparinised capillary tube as soon as possible (and up to a maximum of 3 min) after capture. The time interval from entering the net until completion of blood sampling was recorded to the nearest second (sampling time). Birds were then ringed and biometrics recorded; at Falsterbo this was conducted as part of the Observatory’s standardised monitoring programme. Fat score was collected based on Pettersson and Hasselquist^44^, but adapted for Falsterbo Bird Observatory with classes 0–9 and pectoral muscle scores were recorded following Bairlein et al.^45^. Blood samples were kept cool and spun down with a microhaematocrit centrifuge (CM-70; ELMI Ltd, Latvia) within 15 min of collection. Plasma was aliquoted and immediately frozen. All samples were stored at − 20 °C or below until they were assayed.

### Corticosterone radioimmunoassay

Corticosterone was measured by radioimmunoassay following Wingfield et al. 1992^46^. An exact volume of plasma was equilibrated with 2000 cpm of tritiated corticosterone (purchased from Perkin Elmer, NET399250UC). Steroids were extracted in redistilled dichloromethane, dried under nitrogen in a water bath at 35 °C, and re-suspended in phosphate buffered saline with 1% gelatin. A 100 µl aliquot was added to a scintillation vial and combined with scintillation fluid (Perkin Elmer Ultima Gold: 6013329) to determine percentage recovered from dichloromethane extraction. Duplicate 200 µl aliquots were assayed by adding 100 µl (~ 10^4^ CPM) of tritiated corticosterone (Perkin Elmer NET399250UC) and 100 µl of antibody (MP Biomedical 07–120016, lot 3R3-PB-20E antibody). Unbound steroids were separated from bound steroids with 500 µl of dextran-coated charcoal, followed by centrifugation at 3000 g for 10 min. The supernatant was decanted and combined with scintillation fluid and counted for 5 min or within 2% accuracy on a Beckman 6500 LS counter. Values were corrected for volume and individual recoveries. Inter- and intra-assay variations were 4.45% and 5.50%, respectively, and mean recoveries were 85.1%.

### Statistics

Statistical analyses were conducted in R (version 3.5.3,^13^) and SAS^47^. For all samples and each sample group (Fig. 3), corticosterone concentrations were tested for influence of season (ordinal day) and time of day (time since civil twilight) with Pearson’s product moment correlation coefficient, r (Supplementary Material Fig. S1). As no seasonal or diurnal effects were detected, these parameters were not included in further models. There was little variation in weather conditions at each site as such any influence cannot be tested.

**Figure 4:**
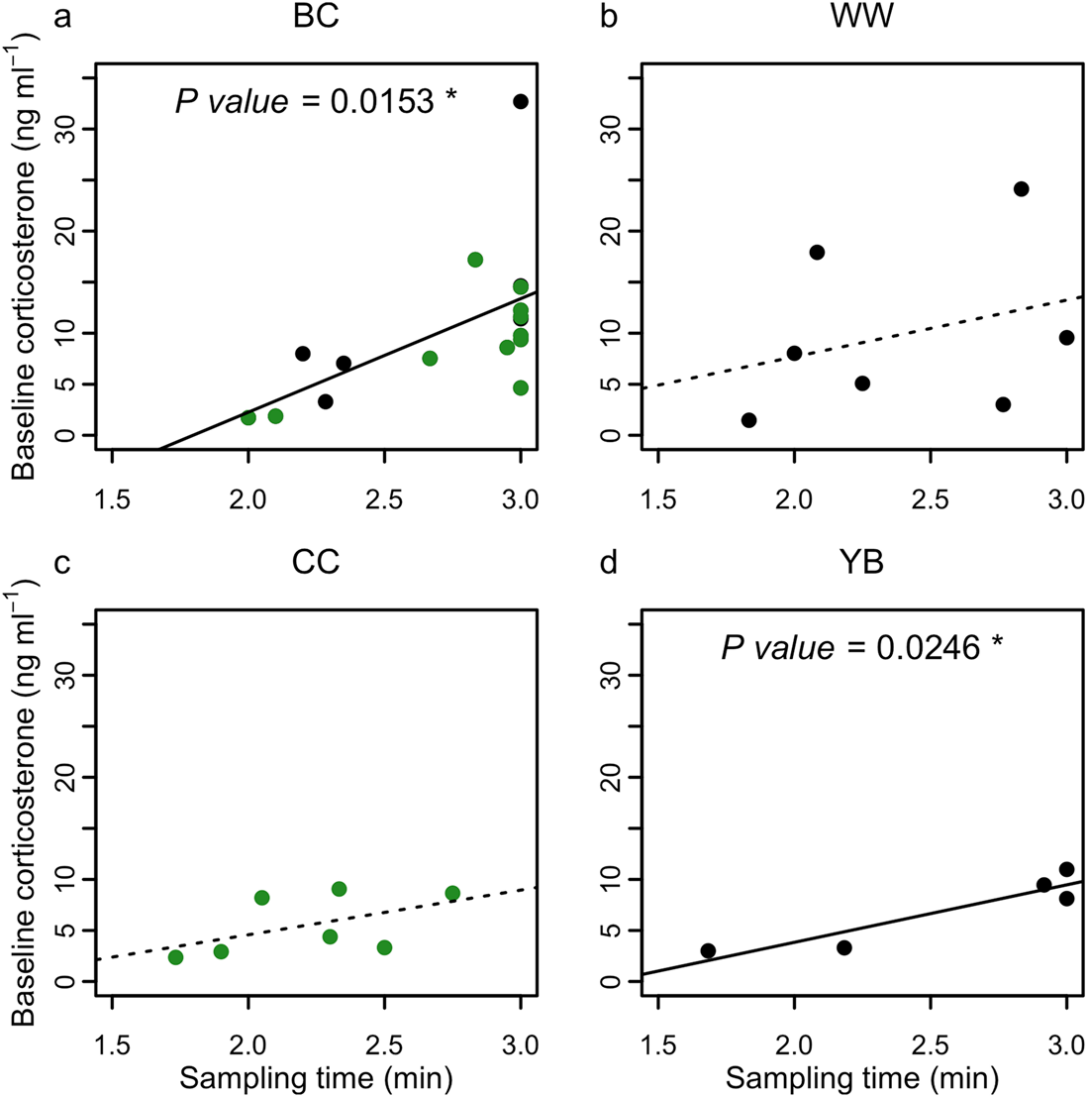
Baseline corticosterone concentrations (ng ml^−1^) and effect of sampling time (minutes post-capture) for all species with multiple samples. (**a**) blackcaps, BC (n = 17); (**b**) willow warblers, WW (n = 7); (**c**) chiffchaffs, CC (n = 7); (**d**) yellow-browed warblers, YB (n = 5). We combined samples from both sites. Lesser whitethroat data (n = 4) is not shown, as all samples were obtained within 6 s. Point colours indicate green: SW controls and grey: SW vagrants.

We a priori tested all samples and samples by species for any effect of the time from capture to sample completion (sampling time) with Pearson’s product moment correlation coefficient, r, and also with Spearman’s rank correlation coefficient, rs, where appropriate. Despite the relatively low sample size, sampling time was evenly distributed across groups. The combined sample showed a positive relationship between corticosterone and sampling time (r = 0.493, *P* = 0.0008). At the species level, this positive relationship was shown for blackcaps (r = 0.577, *P* = 0.0153; rs = 0.698, *P* = 0.0183; Fig. 4a) and yellow-browed warblers (r = 0.577, *P* = 0.0153; Fig. 4d), indicating that sufficient samples were beginning to show above-baseline concentrations (near-baseline) within the 3-min sampling period. The data was assumed to be segmented; however, the linear statistic suggests that there is an influence of the sampling time which must be accounted for in further analysis. There was no support for a sampling time effect in the willow warblers or the chiffchaffs; therefore, for these species we report baseline concentrations of corticosterone (Fig. 4b, c).

Upon a stressor (in this case capture and handling if the bird is not already in an ELHS) corticosterone production is upregulated rapidly. Even within the initial 3 min after capture, if there was an effect of handling corticosterone concentrations were expected to increase exponentially. As such we further tested each dataset for homogeneity of variance (Package ‘car’) and normal distribution of residuals with Shapiro–Wilk Normality Test (Package ‘stats’), plotted and inspected residuals. Where raw rata violated Shapiro–Wilk Normality Test, log transformed values were used in the analysis.

We tested the effect of sample group on corticosterone concentrations with general linear models (GLMs). Backwards-stepwise model refinement method using *P* values was employed to test covariates of the group (migratory group or arrival status) predictor variable. We included the nuisance variable of sampling time in all appropriate models. We tested the relative influence of fat score on corticosterone concentrations, as there was a known relationship between fuel load and adrenal activity. While models have not been overparameterized, the statistical analysis should be interpreted with some caution due to the low sample size.

For the two species with sufficient data (blackcap and willow warblers) we tested the effect of migrant group (SW controls, SW vagrants) on corticosterone concentration, including sampling time and fat score, and the interaction of time × group as parameters. Chiffchaffs were not included in the species specific comparison as all birds were controls. For the complete multispecies dataset we tested the effect of migratory group (SW controls, SW vagrants, SE vagrants), sampling time, and fat and included the interaction parameters of sampling time × group.

Finally, we tested all samples from the Faroes for effect of arrival status (Newly arrived, Other), sampling time and fat score with the interaction parameter arrival status × sampling time. Note that we used linear relationship to conservatively describe the data as a simplified model valid only within the parameter range of the samples. This likely underestimates any expected threshold effect of sampling time or condition score.

Due to our relatively small sample sizes we tested relative effect size for significant models with Cohen’s D test (package ‘emmeans’). Extreme values were tested using Cook’s distance test^48,49^ and Grubb’s outlier test was used if appropriate^50^.

### Compliance with Ethical Standards

All applicable international, national and/or institutional guidelines for the care and use of animals were followed. This study was carried out under permissions from the Copenhagen Bird Ringing Centre, the Natural History Museum of the Faroe Islands, Tórshavn, and Malmö-Lund ethical committee for scientific work with animals, Sweden (M83-16 & M122-14). This study was conducted in accordance to ARRIVE guidelines.

## Results

### Effect of vagrancy, sampling time, and fuel load on corticosterone concentrations

**Figure 5:**
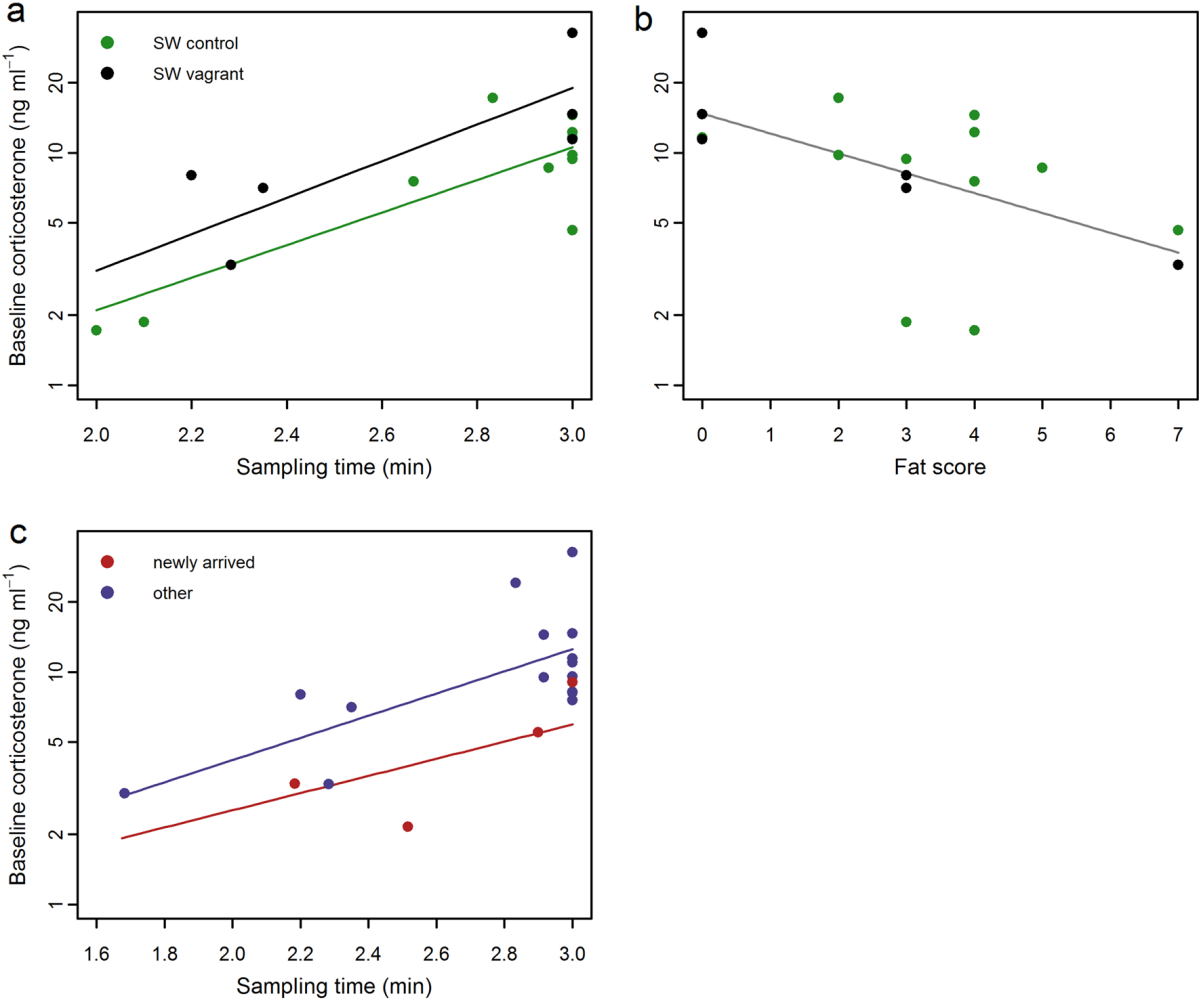
Baseline corticosterone concentrations (ng ml^−1^) and effect of (**a**) sampling time (minutes post-capture) and (**b**) fat score for blackcaps (n = 17); and (**c**) sampling time for all species sampled on the Faroe Islands (n = 20). There was model support for a group effect for SW control birds (green color) and SW vagrant birds (black color) with sampling time. As there was no model support for group effect with fat score, the regression line for the combined dataset is shown as a grey line. Linear relationships for newly-arrived birds (red color) and all others (violet color) with sampling time are supported. Corticosterone concentrations were log transformed in the analysis.

Corticosterone concentrations were generally in the low range expected for baseline levels and ranged between 1.47 and 17.92 ng ml^−1^ in SW controls, 2.16–14.43 ng ml^−1^ in SE vagrants and 3.29–32.69 ng ml^−1^ in SW vagrants (Fig. 3b). One blackcap sample was of a value associated with an ELHS for that species. In species specific comparisons, only blackcaps showed differences in corticosterone concentrations between SW control birds from Falsterbo and SW vagrant birds from the Faroe Islands (sampling time: r = 1.614, *P* < 0.0001 and sampling time × group: r = 0.196, *P* = 0.0363, log transformed; Cohen’s D = 1.20; Fig. 5a) with higher corticosterone in SW vagrant blackcaps and a positive relationship with sampling time. The single high value (32.7 ng ml^−1^; Fig. 2) of a SW vagrant blackcap was significantly out of range (Cook’s D significant, Grubb’s test *P* = 0.001); and equivalent to stress-induced concentrations for autumn migrating blackcaps (mean 23.4 ng ml^−1^; ^42^. When the sample was tested with the ELHS outlier removed, there was no support for a difference in slope (sampling time) between migratory groups (sampling time: r = 8.961, *P* = 0.0017 and sampling time × group: r = 0.672, *P* = 0.3541, natural scale; Table S1). Fuel load, measured by fat score was widely distributed across all samples and ranged between 0 and 7. There were little differences in ranges between migratory groups, and median values were 3 for SW controls, 4 for SE vagrants and 1 for SW vagrants. Corticosterone concentrations in blackcaps correlated negatively with fat scores and correlated positively with sampling time (r = − 0.126, *P* = 0.0306 and r = 1.331, *P* < 0.0001, respectively; log transformed; Table S1, Fig. 5b). The relationship held with the outlier removed (r = − 0.846, *P* = 0.0361 and r = 7.33, *P* = 0.0031, respectively; natural scale; Table S1). We found no additive or interactive effects of fat score or sampling time on corticosterone concentrations in willow warblers (Fig. 2, Table S1). We found no differences in baseline corticosterone concentrations between migratory groups (SW controls, SW vagrants, SE vagrants) in the complete multispecies dataset (Table S1).

### Effect of migratory flight and stopover on corticosterone concentrations

In samples from the Faroes, corticosterone concentrations ranged between 2.16 and 9.04 ng ml^−1^ in birds caught out of migratory flight compared to 3.00–32.69 ng ml^−1^ in all individuals that had been on the islands for an unknown period of time (Fig. 3c). There was a significant difference in circulating corticosterone concentrations between newly arrived migrants and birds who were presumably on the island prior to sampling (sampling time: r = 1.099, *P* < 0.0001 and sampling time × group: r = − 0.249, *P* = 0.0184, log transformed; Cohen’s D = 0.932; Fig. 5c). Newly arrived birds had lower corticosterone concentrations, and despite our low sample size we found a large effect. Fat scores of newly arrived individuals ranged from 0 to 5 and all individuals that had been on the island ranged between 0 and 7; median values were 3 and 4, respectively. Corticosterone concentrations were negatively related to fat scores and positively related to sampling time (r = − 1.49, *P* = 0.0498 natural scale; and r = 1.144, *P* = 0.002, log transformed, respectively; Table S1), but not in the model which combined fat and sampling time (Table S1). The low corticosterone concentration (2.16 ng ml^−1^) of a marsh warbler with a fat score of zero was extreme (outlier with significant leverage, Cook’s D).

## Discussion

We found no evidence to support our prediction of highly elevated corticosterone concentrations as a response to the potentially challenging ecological conditions that occur outside a species normal migratory range. An ELHS could be a way of controlling an immediate facultative behavioural change^51^; but concentrations commensurate with stress-induced values were only recorded in one individual. While we found increased corticosterone synthesis within the first 3 min of capture in SW vagrants on the Faroe Islands compared with controls (SW) migrating along the Fenno–Scandian Flyway, this result appears to be driven by the single outlier who was likely in the ELHS. Body condition, specifically fat score, was negatively correlated with circulating corticosterone concentrations. However, the limited data obtained from newly arrived birds suggests that glucocorticoid synthesis may be suppressed while they are undertaking forced endurance flight.

### Corticosterone and sampling time

We found only limited evidence of an increased rate of corticosterone synthesis across the 3 min sampling period in vagrants compared with controls, which may be expected for improved flexibility to environmental perturbations when outside their normal range^36,52^. Although variation in circulating corticosterone concentration were measured, we did not find high baseline corticosterone concentrations in the range of ELHS levels across the vagrant groups. Baseline measures of corticosterone concentrations were influenced by time until sample completion in two of the four species in samples collected within 3 min. These data are consistent with findings in a meta-analysis across species and seasons^31^, which concluded that samples collected between 2 and 3 min post-capture reflect a near-baseline concentration for the species they considered; as such, sampling time was included as a variable in all analyses. The rate of corticosterone increase from time of capture was likely dependent upon the stage of the annual cycle^31^. In our study, it may indicate that adrenocortical activity is increased such that corticosterone concentrations were rising to detectable levels as soon as 2 min post-capture for the species sampled here.

### Geographic displacement did not elicit an ELHS

Our results indicate that increased adrenocortical activity measured at the baseline sampling point, indicative of an ELHS, did not occur as a physiological reaction to natural displacement in vagrants. Young wind-displaced birds on the Faroe Islands show compensatory orientation in response to navigational error^18^; we infer that such behaviour is not part of a facultative corticosterone stress response, but perhaps integral to the normal migratory processes, despite this being their first migration^1,6,15^. While a larger sample size may indeed show differences between migratory groups taking into account sampling time and fuel stores, this study still has sufficient data to determine that corticosterone concentrations of the amplitude indicative of an ELHS were not generally found in vagrants.

Furthermore, while young birds tracked in Thorup et al.^18^ generally departed in a direction commensurate with corrective behaviour, we cannot rule out the unlikely scenario that the individuals in our study may demonstrate a pioneering phenotype, similar to those classified as SE vagrants, and as such will continue on in their misoriented migratory direction^53^. If out-of-range migration is the norm for pioneers then one would not expect an ELHS associated with a perceived displacement; in contrast, heightened adrenal reactivity may be a beneficial physiological trait in pioneering individuals, which expand the range of the species through long-or short-distance movements^36,54,55^.

### Fuel load and adrenocortical activity

Fat is the primary source of fuel for aerobic activity in birds^21^. And in this study, even in vagrants we found a large range of fat scores, despite the expectation that all vagrants would be lean. Fuel load explained part of the observed variation in baseline corticosterone where lower corticosterone concentrations were related to increased fat scores. Such a relationship with fat score in both baseline and stress-induced corticosterone varies between species and key life history stages in free-living birds^56–61^. Compounding this relationship, weather conditions and fat content interactions result in differential patterns of both baseline and stress-induced corticosterone compared to favourable conditions^62–65^.

Migration itself can affect the dynamics of glucocorticoid synthesis in relation to variation in body condition (fat, mass and muscle). Integration of environmental and internal information involves complex processing, and many studies describe a threshold effect, whereby below a certain stress level glucocorticoid concentrations are not elevated^20^. Based on the *c.* 15-h requisite flight to arrive at the Faroe Islands, one would think that this threshold would be met, but this is dependent upon metabolic reserves at the time of departure from the mainland and remaining fuel load at the time of arrival. For instance, mean baseline corticosterone of ten Western Palearctic migrant species was negatively correlated with mean fat score^24^, and migrating garden warblers with large fuel stores do not elicit an expected ELHS in response to poor foraging opportunities or migratory flight^66^. The threshold for elevations in corticosterone that trigger rapid changes in physiology and behaviour is likely regulated through an integrated measure of body condition^67^. This supports the hunger stress hypothesis^23^ identified in an extremely lean pied flycatcher *Ficedula hypoleuca* caught on migration, which had a baseline corticosterone concentration within the expected range of stress-induced corticosterone. Furthermore, in transiting to an island, birds in poor condition may never arrive thereby our sample is selecting for birds with greater fuel reserves.

One of our baseline titres of circulating corticosterone was high in comparison with other studies^23,24,68^, and within the range of stress-induced values for the same species during autumn migration^42^. This entirely lean blackcap should not be considered a statistical outlier as such, but a biological extreme that was probably experiencing hunger stress. High corticosterone titres can be induced quickly by fasting under controlled conditions. However, increased concentrations are better explained by combined low fat scores and body mass than by duration of fasting^69^. Interestingly, losses in body mass correlate positively with baseline corticosterone, but negatively with stress-induced corticosterone, which may suggest unique regulation of corticosterone signalling during periods of negative energy balance^69,70^. Our data suggest that during the migratory period, severely reduced fuel loads increase corticosterone synthesis, which are associated with aiding with the mobilization of resources, in particular fat and protein, the promotion of foraging and increased night restfulness as an apparent energy saving strategy, although these results are from captive studies^24,71^. The latter behaviour is in direct conflict to the migratory restlessness expected in a nocturnal migrant^33,67^. An ELHS apparently prioritises fuelling while conserving resources by limiting non-essential behaviours, including, potentially, the continuation of migration^72^. There are instances when challenging conditions and reduced body condition do not affect baseline corticosterone (although the variance does increase), but can promote substantial changes in stress-induced corticosterone^62,63,73^. A stress-induced corticosterone titre was not obtained in this study, primarily due to the extremely small body size of the study species. Studies of this design suggest that sufficient energy reserves are still available to fuel metabolism in the face of a single environmental challenge, but that the system up-regulates corticosterone synthesis when faced with an additional challenge. This may be important for improving the rate at which the organism can respond to an environmental challenge^52,74^.

### Endurance flight and corticosterone

Contrary to our predictions of elevated corticosterone, we found significantly reduced concentrations of circulating corticosterone in birds apparently caught out of endurance migratory flight compared to birds that had previously arrived on the island. The extremely low baseline titre in a newly arrived marsh warbler completely devoid of visible fat was unexpected (see previous section). Exhaustion of fuel reserves is expected to trigger an increase in corticosterone production, and in the overall sample higher corticosterone concentrations are associated with lower fat scores. This suggests that prolonged obligate flight may initiate feedback mechanisms that suppress the HPA axis in critically lean migrants^75^. Such a response may be adaptive during water crossings, where it clearly pays to continue flight for as long as physiologically possible. Alternatively, adrenocortical activity may be suppressed to protect the skeletal muscle essential for flight, which is catabolised by chronic high concentrations of plasma corticosterone^76^. It cannot be determined whether this occurs in all cases or only when up-regulation of the HPA activity resulting in increased corticosterone may be expected (here, as a response to depleted fuel reserves). We caveat that this observation must be interpreted with caution as it is based on a single individual. Previous studies in passerines, homing pigeons and waders, in which birds are captured out of migratory flight or immediately after landing, reported increased concentrations of baseline corticosterone in comparison with resting birds^56,77,78^. These field studies were generally conducted at inland sites where it was impossible to determine where the birds initiated their migration^23,56^. The same is true for wind tunnel experiments in which the subjects were able to discontinue the flight at will: differential results were obtained after flights of different durations^79^. The most analogous study was conducted on birds recently arrived after the spring Mediterranean crossing (a similar water crossing distance); however, while most of the birds sampled were very recent arrivals, the baseline corticosterone profile was more representative of our overall pattern of corticosterone concentrations in all birds on the island, rather than the low values of newly arrived individuals^24^. This could potentially be explained by sampling time (many were obtained between 3 and 6 min post-capture) or because the birds were not captured directly out of migratory flight. A robust sample size in our study would be necessary to provide any certainly that we can discount that a negative relationship between fat and corticosterone prevails or is even greater in birds caught out of endurance flight under natural conditions. Furthermore, the occurrence of birds captured immediately following and carrying substantial fuel store is noteworthy in itself.

### Outlook

The study of vagrancy is both nuanced and difficult due to inherently low sample sizes. Yet, given the global effects of climate change, it is highly likely that aberrant behaviours such as vagrancy will become more widespread and frequent. This study, although limited, yielded observations of low stress levels when high stress would be expected under certain critical circumstance and challenges the dogma of stress associated with long-distance migration and birds outside their normal range. Understanding how birds endure such vagrancy, in addition to the suite of known challenges, may have conservation implications. This study outlines some potential pathways by which vagrancy affects migrant birds, highlighting fields that future research should address.

## Supplementary Information

Supplementary Information.

## Data Availability

All raw data files generated during this study are available from the corresponding author on reasonable request.
