# Supplementary material for: Integrating orientation mechanisms, adrenocortical activity, and endurance flight in vagrancy behaviour
Source: Sci Rep. 2022 Dec 21;12:22104. doi: 10.1038/s41598-022-26136-8 (PMC9772197; doi:10.1038/s41598-022-26136-8)
Supplement: Supplementary file 1 — Supplementary Information. [file 41598_2022_26136_MOESM1_ESM.pdf]

Supplementary Material

Integrating orientation mechanisms, adrenocortical activity and endurance flight in vagrancy behaviour

Scientific Reports

Katherine R. S. Snell*, Rebecca C. Young, Jesse S. Krause, J. Martin Collinson, John C. Wingfield & Kasper Thorup

*Corresponding author: ksnell@ab.mpg.de Center for Macroecology, Evolution and Climate, Natural History Museum of Denmark, University of Copenhagen, Universitetsparken 15, 2100 Copenhagen, Denmark

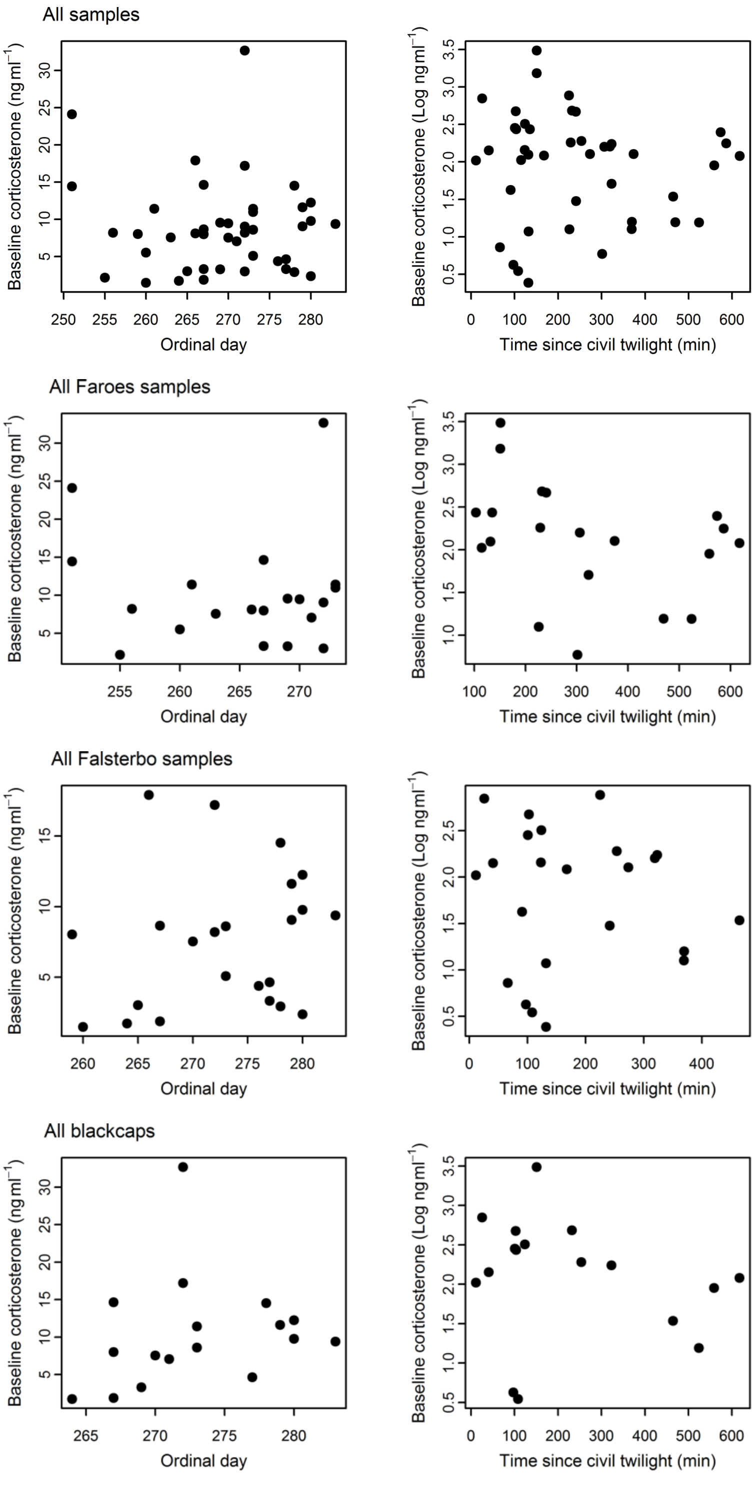

Fig. S1 Corticosterone concentration measures (ng ml^{-1}) by season (ordinal day) and by location-corrected time of day (time since civil twilight (min)) for all sample groups (All samples: all species all sampling locations; All samples from the Faroes: both SW vagrants and SE vagrants; All samples from Falsterbo: SW control; and All blackcaps: both SW vagrants and SW controls). No linear relationship was detected for season or time of day.

Table S1 General linear models with backwards stepwise model refinement method to test covariates of the group (migratory group or arrival status) and fat score as predictor variables. We included the nuisance variable of sampling time in all appropriate models. Data tested as natural and log transformed. The models were tested for normal distribution of residuals with Shapiro-Wilk Normality Test and where violated the output is given in grey italics. Best fit models are given in bold.

	Independent variables			natural scale			*Estimate, F* and *P*-values for each variable		log-transformed			*Estimate* and *P*-values for each variable	
**Data**	**IP1**	**IP2**	**IP3**	**Adj R-squared**	**shapiro**	***r* 1; F1; *P* 1**	***r* 2; F2; *P* 2**	***r* 3; F3; *P* 3**	**Adj R-squared**	**shapiro**	***r* 1; F1; *P* 1**	***r* 2; F2; *P* 2**	***r* 3; F3; *P* 3**
**BC**	Time	Time×Origin	Fat	*0.4811*	***0.06409***	*9.444; 6.3448; 0.0256*	*1.414; 1.643; 0.222*	*-1.138; 2.711; 0.1235*	0.6964	0.5825	1.4336; 22.533; 0.000381	0.1341; 2.280; 0.154904	-0.090; 2.616; 0.129765
	Time	Time×Origin		*0.4177*	*0.03916*	*11.727; 10.0981; 0.00671*	*2.196; 4.3326; 0.0562*		**0.6614**	**0.9905**	**1.6142; 29.669; <0.001**	**0.1960; 5.355; 0.03635**	
	Time	Fat		***0.4573***	*0.007857*	***8.36; 5.0104; 0.0420***	***-1.52; 5.6699; 0.0320***		**0.6687**	**0.3914**	**1.3310; 18.7441; 0.000692**	**-0.1262; 5.780; 0.03061**	
a priori	**Time**			***0.2883***	*0.001173*	***11.125; 7.4811; 0.01534***			**0.5631**	**0.7408**	**1.5604; 21.619; 0.000314**		
**BCx**	Time	Time×Origin	Fat	0.5742	**0.1279**	7.505; 11.903; 0.00480	0.2066; 0.094; 0.76345	0.8040; 4.036; 0.06756	0.6535	0.3716	1.36858; 20.9493; 0.00063	0.09369; 1.0316; 0.3298321	-0.07883; 2.0540; 0.1773450
	Time	Time×Origin		0.4748	**0.4435**	8.9610; 15.472; 0.00171	0.672; 0.923; 0.35410		0.6254	0.9224	1.51131; 26.5794; 0.0001849	0.13935; 2.3964; 0.1456053	
	Time	Fat		**0.6039**	**0.1525**	**7.3271; 13.123; 0.00309**	**-0.8466; 5.461; 0.03609**		0.6526	**0.911**	1.28775; 19.913; 0.00063	-0.098; 3.604; 0.0800	
a priori	**Time**			**0.6039**	**0.463**	**8.587; 14.7155 ; 0.00182**			**0.588**	**0.9737**	**1.4338 ; 22.4071 ; 0.00032**		
**WW**	Time	Time×Origin	Fat	-0.2387	0.5945	-5.23; 0.3501; 0.596	4.322; 1.057; 0.3795	-1.506; 0.1754; 0.7035	*-0.4018*	*0.9379*	*-0.27393; 0.5141; 0.5251*	*0.4353; 0.6754; 0.4714*	*-0.03472; 0.0059; 0.9437*
	Time	Time×Origin		0.01668	0.3991	-5.295; 0.5872; 0.4862	4.565; 1.5146; 0.2859		*-0.05341*	*0.977*	*-0.2754; 0.7558; 0.4337*	*0.4409; 0.9400; 0.3872*	
	Time	Fat		-0.2564	0.4026	4.865; 0.3452; 0.5884	-2.016; 0.3161; 0.6040		*-0.288*	*0.6597*	*0.74288; 0.5596; 0.496*	*-0.08614; 0.0401; 0.851*	
	Origin	Fat		0.0387	0.1215	9.046; 1.6790; 0.2648	-1.385; 0.1889; 0.6863		*-0.04745*	*0.9656*	*1.09418; 1.6069; 0.2737*	*-0.01963; 0.0025; 0.9627*	
	Origin			0.1947	0.4264	9.738; 2.4503; 0.1783			*0.1615*	*0.9428*	*1.1040; 2.1558; 0.202*		
a priori	Time			-0.08453	**0.2264**	5.552; 0.5324; 0.4983			*-0.04077*	*0.4603*	*0.77223; 0.7649; 0.4218*		
**All**	Time	Time×Origin	Fat	*0.3113*	*0.0007123*	*6.0719; 9.4327 ; 0.00393*	*-0.3291(E) 1.2961(W); 1.8873; 0.65330(E) 0.10852(W)*	*-0.6043; 1.7414; 0.19486*	0.4078	0.4253	1.01105; 21.7876 ;<0.0001	-0.04123(E) 0.10237(W); 1.1519 ; 0.608(E) 0.243(W)	-0.03482; 0.4816 ; 0.492
	Time	Time×Origin		*0.2982*	*0.0004321*	*6.4095; 10.4907; 0.00245*	*-0.4682(E) 1.5507 (W); 3.1426 ;0.52282(E) 0.05150 (W)*		0.4157	0.2701	1.03051; 23.3300 ; <0.0001	-0.04924(E) 0.11703(W); 1.7593 ; 0.533(E) 0.168(W)	
	Time	Fat		***0.2807***	*<0.001*	***6.3535; 11.7495; 0.00142***	***-0.9004; 4.1858; 0.04738***		0.4033	0.7334	1.02053; 26.1749; <0.0001	-0.06060; 1.6371 ; 0.208	
a priori	Time			***0.2248***	*<0.001*	***6.911; 13.182; 0.00077***			**0.3941**	**0.4813**	**1.0580; 28.3138; <0.001**		
**Faroes**	Time	Time×Arr	Fat	*0.2969*	*0.008167*	*6.88; 3.559; 0.775*	*-1.878; 2.131; 0.167*	*-1.0194; 2.281; 0.1504*	0.5417	0.8629	1.03215; 0.731; 0.40494	-0.2395; 6.1392; 0.024757	0.0434; 0.731; 0.40494
	Time	Time×Arr		*0.2439*	*0.0001828*	*8.446; 5.6375; 0.0296*	*-2.092; 2.4904; 0.1329*		**0.5489**	**0.3139**	**1.09882; 16.8328; 0.000742**	**-0.24871; 6.8043; 0.018354**	
	Time	Fat		*0.2501*	*0.01912*	*7.0485; 3.3371; 0.0853*	*-1.128; 2.6514; 0.12185*		0.4031	0.4194	1.05366 ; 10.8078; 0.0043	-0.05726 ; 0.9902; 0.333658	
	Fat			***0.1527***	***0.1109***	***-1.4857; 4.424; 0.0497***			0.07787	0.5729	-0.11072; 2.6045; 0.124		
a priori	Time			***0.1813***	*0.0002248*	***8.824; 5.2064; 0.03489***			**0.4034**	**0.6497**	**1.1438; 13.8490; 0.001562**		

Adjusted R-squared vaules are given for each model and the estimate, F and P-values for each variable. Data sets for testing the effect of (i) migratory group on corticosterone concentrations are as follows: BC - all blackcap data; BCx - Blackcap data excluding the ELHS outlier; WW - all willow warbler data; All - all data. We report the origin statistic of the vagrant group relative to the control group, in the case of All data the SE and SW vargant groups are denoted by (E) and (W) respectively. For testing the effect of (ii) arrival status (Arr) on corticosterone concentrations we used all data from the Faroes. We report the arrival status statistic of the Newly arrived group relative to the Others. For each data set we a priori *tested the effect of time to indicate if this variable must be included in all models.*
